# Effectiveness of triple therapy with direct-acting antivirals for hepatitis C genotype 1 infection: application of propensity score matching in a national HCV treatment registry

**DOI:** 10.1186/s12913-017-2188-1

**Published:** 2017-04-19

**Authors:** Emma Gray, David J. Pasta, Suzanne Norris, Aisling O’Leary, on behalf of the Irish Hepatitis C Outcomes and Research Network (ICORN)

**Affiliations:** 1 School of Medicine, Trinity College Dublin, Dublin, Ireland; 2 ICON Clinical Research, San Francisco, CA USA; 3 Department of Hepatology, St James’ Hospital, Dublin, Ireland; 4 National Centre for Pharmacoeconomics, St. James’ Hospital, Dublin, Ireland; 5 School of Pharmacy, Royal College of Surgeons of Ireland, Dublin, Ireland

**Keywords:** Comparative effectiveness, Propensity score matching, Outcomes Research, Protease inhibitor, Telaprevir, Boceprevir, Sustained virological response

## Abstract

**Background:**

Observational studies are used to measure the effectiveness of an intervention in non-experimental, real world scenarios at the population level and are recognised as an important component of the evidence pyramid. Such data can be accrued through prospective cohort studies and a patient registry is a proven method for this type of study. The national hepatitis C (HCV) registry was established in Ireland in 2012 with the aim of monitoring the clinical and economic outcomes from new, high cost regimens for the treatment of HCV infection. A sustained virological response (SVR) 24 weeks following completion of therapy with interferon-containing regimens is considered a cure. Non-randomisation in these studies can result in confounding or selection bias. Propensity score (PS) matching is one of a number of statistical tools that can be used to mitigate the effects of confounding in observational studies.

**Methods:**

We analysed the data of 309 patients who underwent triple therapy treatment with telaprevir (TPV) in combination with pegylated-interferon and ribavirin (PR) or boceprevir (BOC)/PR between June 2012 and December 2014. The decision to initiate treatment and the selection of the treatment regimen was at the discretion of the physician. To adjust for confounding, three approaches to propensity score matching were assessed Adjusted sustained-virological response rates (SVR), odds ratios, *p*-values and 95% confidence intervals were calculated from the three PS matched dataset.

**Results:**

Prior to matching, the unadjusted sustained virological response rates 24 weeks after treatment complete (SVR24) were 74% (*n* = 158/215) and 61% (*n* = 57/94) for telaprevir/PR and boceprevir/PR, respectively. After matching, adjusted SVR24 rates were between 73–74% and 60–61% for telaprevir/PR and boceprevir/PR, respectively.

**Conclusion:**

Efficacy rates were comparable with those reported in pivotal clinical trials and real world studies. After adjusting for confounding, we conclude that there was no difference in treatment effect after PS matching. The small sample size limits the conclusions that can be made about the effect of PS matching. Propensity score adjustment remains a tool that can be applied to future analysis, however, we suggest, where possible, using a larger sample size in order to reduce the uncertainty around the outcomes.

**Electronic supplementary material:**

The online version of this article (doi:10.1186/s12913-017-2188-1) contains supplementary material, which is available to authorized users.

## Background

There is on-going debate about the merits of using observational evidence either to estimate relative treatment effect in the absence of randomised evidence or as an adjunct to it [1–3]. While considered the ‘gold’ standard in the hierarchy of research designs for evaluating the efficacy and safety of treatment interventions, the value of relying on randomised controlled trials (RCTs) for estimating treatment effectiveness in the clinical setting is limited [4–6]. This arises from the strict inclusion and exclusion criteria in these trials, with results which may have limited applicability to patients in real-world clinical settings [7, 8].

Observational research is becoming increasingly recognized as an important component of the evidence pyramid, as it can provide valuable information regarding the effectiveness and appropriate use of agents in the real-world, outside of clinical trials [2, 9, 10]. A comprehensive evidence base, including both RCTs and high-quality, well-designed observational studies, is important and can enhance reimbursement decision providing decision-makers with a greater evidence-base from which to make their assessments [4, 6, 11]. The potential for registries in collecting real world data is substantial [12]. However, the major limitation to this study type is the lack of randomisation to allocate, by chance, the risk factors for an outcome of interest [13]. The process of randomisation ensures that subjects are allocated to treatment or comparator groups by chance [14]. Absence of random allocation in observational studies leads to a lack of internal validity and can result in confounding [4, 15–21].

Confounding is a form of bias that occurs when one or more variables or risk factors influences the outcomes of interest and therefore, impacts the true measure of association [22]. The non-randomised nature of observational research studies increases their susceptibility to confounding bias. While in RCTs, confounding variables are balanced during the study design phase, the adjustment of confounders in observational studies is completed during the analysis phase [23]. Propensity score (PS) matching is one of a number of approaches that have been developed to reduce confounding. It involves the generation of a score that summarises the confounding by multiple variables. PS matching involves the formation of matched pairs of treated and untreated subject. The pairs are formed between subjects with similar PS values. There are a number of different approaches to PS matching but nearest neighbour matching without replacement within a specified caliper limit of the PS is the most common. This approach will be applied to the PS matching in this thesis [24–27]. The use of a caliper limit ensures that the difference in the PS score between the matched pairs lies within this specified distance. Naïve matching is also possible, where no limit is placed on the caliper width. All treated subjects are matched to untreated subjects until every subject has been matched. This method makes the assumption that the distribution of baseline covariates is similar within a matched pair.

Infection with hepatitis C virus (HCV) is a major public health problem and a leading cause of chronic liver disease [28]. It is estimated to affect >185 million people, with a prevalence of approximately 3% in the worldwide population [29]. In Ireland, HCV is estimated to affect between 20,000 and 50,000 people [30]. Over the last twenty years, treatment for HCV has evolved rapidly. Since 2011, with the development of the novel, first generation directly acting antiviral (DAA) agents telaprevir (TPV) and boceprevir (BOC), for use in combination with pegylated-interferon and ribavirin (PR), changed the optimal treatment regimen for genotype (GT) 1 HCV infection [31]. However, these treatment regimens are complex and associated with significant side effects, necessitating intensive on-treatment monitoring. Therefore, the capacity to treat patients in the seven hepatology and infectious disease treatment units in Ireland has been limited.

Given the high drug acquisitions cost of these agents, epidemiological data indicates the budget impact of treating the HCV-infected cohort in Ireland to be significant and raises the question of affordability [32]. The approval of these two DAA regimens in Ireland In 2012 was recognized as an opportune time to maximize the therapeutic management of HCV infection in Ireland. Thus, a national HCV treatment registry was established to ensure optimal clinical and economic outcomes from the use of new agents to the market.

The aim of this study is to determine the clinical outcomes from the national HCV treatment registry for patients treated with TPV/PR or BOC/PR and to assess the application of PS matching in a national registry.

## Methods

The Irish national HCV treatment registry utilises a prospective, longitudinal, observational methodology. Since 2012, patients prescribed HCV DAA treatment have been enrolled for participation in the registry. For this study, we analysed the data of 309 patients between June 2012 and December 2014. Patients were eligible if they were HCV GT1 infected and 18 years or older. Cirrhotic and non-cirrhotic patients, those with or without a history of previous HCV treatment exposure and HIV co-infected patients were eligible for inclusion.

The decision to initiate treatment and the selection of the treatment regimen was at the discretion of the physician. Patients were treated with either:

- Telaprevir in combination with pegylated interferon and ribavirin (TPV/PR)
- Boceprevir in combination with pegylated interferon and ribavirin (BOC/PR)

Demographic and clinical data were collected at baseline, throughout treatment and in the post-treatment follow-up period. Treatment effectiveness was measured as sustained virological response 24-weeks post treatment completion (SVR24). SVR24 was defined as HCV-RNA level below the level of quantification or undetected recorded at least 24 weeks after cessation of treatment. Where a SVR was not achieved, the frequency of virological failure and relapse were recorded. Additionally, patients who were lost-to-follow up or had discontinued treatment prematurely as a result of adverse events, non-compliance or other factors were captured.

### Statistical analysis

Descriptive statistics were used to present the baseline characteristics and unadjusted outcomes for the study population. Categorical variables were reported as frequencies and percentages. Baseline continuous data were expressed as medians (with the interquartile range (IQR)). Univariate analyses were performed using Chi-square, Fisher’s Exact or Student’s *t*-test as appropriate. The SVR24 was measured on an ‘intent-to-treat’ basis with all patients starting treatment contributing to the denominator.

The numbers of patients included in the study were limited by the capacity to treat in the designated treatment clinics in Ireland at the time. We included all patients who received treatment with TPV/PR or BOC/PR in the defined period of the study which was 309. This number does not have sufficient power to demonstrate statistical significance. Therefore, conclusions about the statistical significance and treatment effect are therefore, limited.

The PS, the probability of being treated with TPV/PR, as opposed to BOC/PR, given other known baseline demographics and HCV characteristics, was computed using logistic regression. Following a review of the literature and consultation with clinicians, nine covariates were entered into the model: previous treatment experience, presence of cirrhosis, age, BMI, GT1, GT1b, IL28B CT, IL28B TT and baseline HCV > 800,000 IU/ml [33–37]. For both genotype and IL28B allele, both of which had more than two distinct categories (i.e., GT1 (no subtype), GT1a, GT1b), dummy variables were created to represent these subgroups in the regression analysis (Additional file 1: Tables S1 and S2). The resultant PS (range = 0.0–1.0) was a single score per patient, with a high score representing a high probability that the patient would be treated with TPV/PR, based on the given information. Prior to applying PS matching, a comparison of confounders between the TPV/PR and BOC/PR groups was completed. The standardised difference was used to compare the mean of continuous and binary variables between treatment groups and is not influenced by the sample size.

Three approaches to nearest neighbor matching without replacement were employed to match patients who received TPV/PR with patients who received BOC/PR. A treated subject (TPV/PR) was first selected at random. The untreated subject (BOC/PR) whose PS was closest to that of this randomly selected treated subject was chosen for matching. This process was then repeated until untreated subject have been matched to all treated subjects. In our first approach, naïve matching was used (no limit was placed on the caliper width) whereby all ninety-four BOC/PR subjects were matched to a TPV/PR subject. A second matching approach was completed where patients were matched on the logit of the PS using a caliper width equal to 0.1 of the logit of the PS. Finally, in the third scenario, the caliper width was extended to 0.2 of the logit of the PS. In all three approaches, each TPV/PR treated subject was matched to the BOC/PR subject with the closest PS. Adjusted SVR rates, odds ratios (OR), *p*-values and 95% confidence intervals (CI) were calculated.

Demographic and outcome analyses and multiple imputation were conducted using SPSS Version 21 (IBM Corp, Armonk, NY, USA) [38]. PS matching was conducted with STATA Version 14 (STATACorp, College Station, Texas, USA) [39].

## Results

A total of 309 patients with HCV infection initiated treatment with TPV/PR (*n* = 215) or BOC/PR (*n* = 94). All have reached SVR24 (*n* = 222/309) or discontinued treatment prematurely (*n* = 87/309). The majority of patients were male (73.1%) with a median age of 46 years (IQR 38–54 years). Patients who previously failed to respond to treatment with PR accounted for 29.1% of the cohort while cirrhosis was present in 27.1%. HIV co-infection accounts for 7.3%. IL28B CT was the dominant (53.5%) allele. The proportion of patients with GT1a, 1b and 1 (no subtype) was 55.7, 27.8 and 16.5% respectively. Baseline HCV-RNA was greater than 800,000 IU/ml in 53.5% of the cohort (Table 1).

**Table 1:** Baseline demographics of the cohort

	Total Cohort	TPV/PR	BOC/PR
	*n* = 309	*n* = 215	*n* = 94
Age – Years, Median (IQR)	46 (38–54)	45 (38–54)	47 (39–56)
BMI – kg/m^2^ Median (IQR)	26.5 (23.5–28.8)	26.3 (23.9–28.5)	25.9 (21.4–30.6)
Male, n (%)	212/290 (73.1%)	149/202 (73.8%)	63/88 (71.6%)
History of cirrhosis, n (%)	79/291 (27.1%)	55/198 (27.8%)	24/93 (25.8%)
Treatment Experienced, n (%)	89/306 (29.1%)	66/212 (31.1%)	23/94 (24.5%)
HIV co-infected, n (%)	23/313 (7.3%)	23/198 (11.6%)	–
IL28B Allele, n (%)			
CC	92/269 (34.2%)	59/179 (33%)	31/86 (36%)
CT	144/269 (53.5%)	96/179 (53.6%)	47/86 (54.5%)
TT	33/269 (12.3%)	24/179 (13.4%)	8/86 (9.3%)
Genotype, n (%)			
G1a	172/309 (55.7%)	119/215 (55.3%)	53/94 (56.4%)
G1b	86/309 (27.8%)	55/215 (25.6%)	31/94 (33%)
G1 – unspecified	51/309 (16.5%)	41/215 (19.1%)	10/94 (10.6%)
Acquisition Risk Factor			
IVDU	133/309 (43%)	89/215 (41.7%)	44/94 (46.8%)
Anti-D	28/309 (9.1%)	18/215 (8.4%)	10/94 (10.6%)
Blood product	36/309 (11.7%)	31/215 (14.4%)	5/94 (5.3%)
Other	15/309 (4.9%)	11/215 (5.1%)	4/94 (4.4%)
Unknown/not reported	97/309 (31.4%)	66/215 (30.7%)	31/94 (32.9%)
Baseline HCV-RNA >800,000 IU/ml	153/286 (53.5%)	98/196 (50%)	55/90 (61.1%)

Missing data is a common problem with observational data. Percentages are calculated based on the proportion of available data

### Effectiveness

**Fig. 1:**
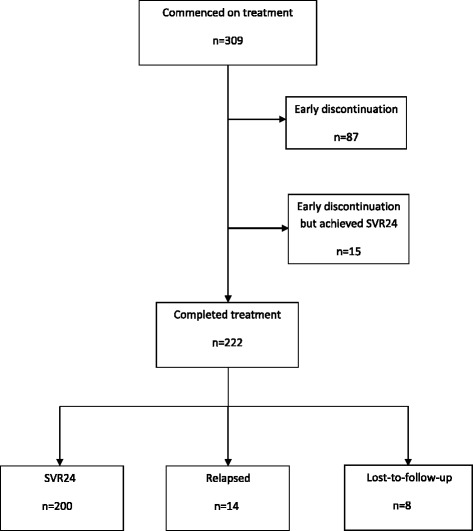
Flow diagram illustrating the outcomes of patients ever started on treatment with TPV/PR and BOC/PR. ^#^Fifteen patients discontinued treatment prematurely but achieved a SVR24. Therefore, in total *n* = 215/309 (70%) (*n* = 200/222 and *n* = 15/87) achieved a SVR24

Outcomes for all 309 patients are presented in Table 2. Overall, 72% (*n* = 222/309) of the cohort completed therapy, with the remaining 28% (*n* = 87/309) discontinuing early due to adverse events (AEs), virological failure, poor tolerability, non-compliance or for an undetermined reason. The overall rate of SVR24 (unadjusted) was 70% (*n* = 215/309). This included fifteen patients who discontinued prematurely as a result of an AE or non-compliance but achieved an SVR24. Fourteen patients (5%) completed a full course of treatment but relapsed within 24-weeks of completing therapy. There were eight patients (2%) considered lost to follow-up. These patients completed a full course of therapy but failed to return to the clinic for any SVR assessment (Fig. 1).

**Table 2:** Unadjusted SVR rates among patients stratified by treatment choice and baseline HCV characteristics

	Total *N* = 309			TPV/PR *N* = 215			BOC/PR *N* = 94		
	n/N	SVR12	95% CI	n/N	SVR12	95% CI	n/N	SVR12	95% CI
Overall	215/309	69.6	64.5–74.7	158/215	73.5	67.6–79.4	57/94	60.6	50.7–70.5
Absence of cirrhosis	158/212	74.5	68.6–80.4	113/143	79	72.3–85.7	45/69	65.2	54–76.4
Presence of cirrhosis	48/79	60.8	47.8–69.2	36/55	65.5	52.9–78.1	12/24	50	30–70
Treatment naïve	153/217	70.5	64.4–76.6	109/146	74.7	67.6–81.8	44/71	56.5	36.2–76.4
Treatment experienced	61/89	68.5	58.8–78.2	48/66	72.7	62–83.4	13/23	62	50.7–73.3
Genotype 1a	115/172	66.9	59.9–73.9	84/119	70.6	62.4–78.8	31/53	58.5	45.2–71.8
Genotype 1b	64/86	74.4	65.2–83.6	46/55	83.6	78.8–93.4	18/31	58.1	40.7–70.5
Genotype 1 (no subtype)	35/49	71.4	58.7–84.1	27/39	69.2	54.7–83.7	8/10	80	55.2–104.8

**Fig. 2:**
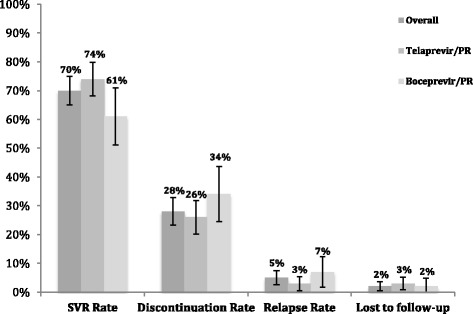
Unadjusted treatment outcomes for the overall cohort and stratified per treatment regimen. # Fifteen patients discontinued treatment prematurely but achieved an SVR24; eleven patients treated with TPV/PR and four patients treated with BOC/PR. These patients were counted in both the discontinuation rate and the SVR rate

The unadjusted SVR24 rates were 74% (*n* = 158/215) and 61% (*n* = 57/94) for TPV/PR and BOC/PR, respectively. Discontinuation and relapse rates were comparable between the two treatments (Fig. 2). Prior to adjusting for confounding, the crude odds of SVR in patients treated with TPV/PR were 80% greater than those treated with BOC/PR (OR = 1.8, 95% CI 1.08–3, *p* = 0.025).

The unadjusted SVR24 rates varied according to the presence or absence of baseline cirrhosis, prior treatment experience and GT1 subtype (Table 2). In the absence of cirrhosis, the SVR24 was 79% (*n* = 113/143) for patients treated with TPV/PR and 65% (*n* = 45/69) for patients treated with BOC/PR. Presence of cirrhosis led to SVR24 rates of 66% (*n* = 36/55) and 50% (12/24) in TPV/PR and BOC/PR cohorts, respectively.

The SVR24 for treatment naïve patients treated with TPV/PR was 75% (*n* = 109/146) and 57% (*n* = 44/71) for patients who received BOC/PR. Previous treatment experience resulted in SVR24 rates of 73 and 62% in those treated with TPV/PR and BOC/PR respectively. The SVR24 rates were higher in GT1a and GT1b patients treated with TPV/PR compared with those treated with BOC/PR (71% and 84% vs. 59% and 58% for GT1a and GT1b TPV/PR and BOC/PR, respectively).

### Propensity score matched analysis

Prior to applying PS matching, a comparison of confounders between the TPV/PR and BOC/PR groups was completed. The standardised difference of confounding variables prior to matching is presented in Table 3. Variables with a standardised difference greater than 0.1 exhibit imbalance between the two treatment groups.

**Table 3:** Standardised difference of confounding variables between TPV/PR and BOC/PR patients prior to matching

	Mean TPV/PR	Mean BOC/PR	Standardised difference
Treatment experinced	0.31	0.24	0.154
Presence of cirrhosis	0.29	0.26	0.058
Age	45.73	47.62	−0.174
BMI	26.7	26.73	−0.006
Baseline HCV > 800,000	0.49	0.6	−0.203
GT1	0.19	0.11	0.225
GT1b	0.26	0.33	−0.153
IL28B CT	0.49	0.53	−0.072
IL28B TT	0.17	0.11	0.119

#### Naïve matching

After matching, ninety-four matched pairs were formed. The standardised difference of confounding variables after naïve matching shows improvements in balance between the two groups (Table 4).

**Table 4:** Standardised difference of confounding variables between TPV/PR and BOC/PR patients after the three approaches to matching

	After naïve matching	After 0.1 caliper limit	After 0.2 caliper limit
Treatment experinced	0.000	0.04	0.04
Presence of cirrhosis	0.000	0.022	0.022
Age	−0.014	0.011	0.016
BMI	−0.003	−0.005	−0.002
Baseline HCV > 800,000	0.019	0.019	0.019
GT1	−0.022	0.000	0.000
GT1b	0.019	0.039	0.039
IL28B CT	0.018	−0.018	−0.018
IL28B TT	0.027	0.072	0.028

The adjusted SVR rates were 73% (*n* = 69/94) and 61% (*n* = 57/94) for TPV/PR and BOC/PR, respectively. The adjusted odds of SVR in patients treated with TPV/PR were 76% greater than those treated with BOC/PR (OR = 1.76, 95% CI 0.868–3.58, *p* = 0.116).

#### Matching with a 0.1 Caliper Limit

After matching, applying a caliper width limit of 0.1, ninety matched pairs were formed. Four pairs were excluded after the caliper limit was applied due a difference in the logit of the PS being greater than 0.1. The confounding variables after matching shows greater balance than prior to matching (Table 4).

The adjusted SVR rates were 73% (*n* = 66/90) and 60% (*n* = 54/90) for TPV/PR and BOC/PR, respectively. The adjusted odds of SVR in patients treated with TPV/PR were 87% greater than those treated with BOC/PR (OR = 1.87, 95% CI 0.89–3.95, *p* = 0.097).

#### Matching with a 0.2 Caliper Limit

After adjusting the caliper width limit to 0.2, ninety-one matched pairs were formed. Three pairs were excluded after the caliper width limit of 0.2 was applied. After matching, the standardised difference for all covariates is less than 0.1 (Table).

The adjusted SVR rates were 74% (*n* = 67/91) and 60% (*n* = 57/91) for TPV/PR and BOC/PR, respectively. The adjusted odds of SVR in patients treated with TPV/PR were 85% greater than those treated with BOC/PR (OR = 1.85, 95% CI 0.883–3.88, *p* = 0.102).

**Fig. 3:**
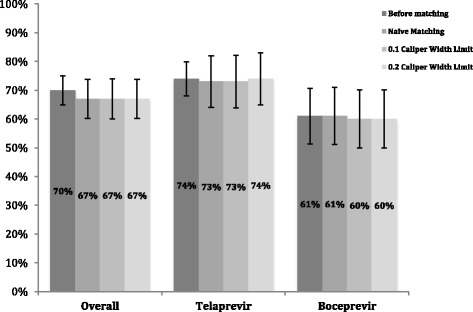
Adjusted SVR rates after propensity score matching for TPV/PR and BOC/PR treated patients

Figure 3 illustrates the SVR24 rates for TPV/PR and BOC/PR prior to and after applying the PS matching methodology. There is no significant change in the treatment effect after PS matching.

### Comparison with clinical trial data

**Fig. 4:**
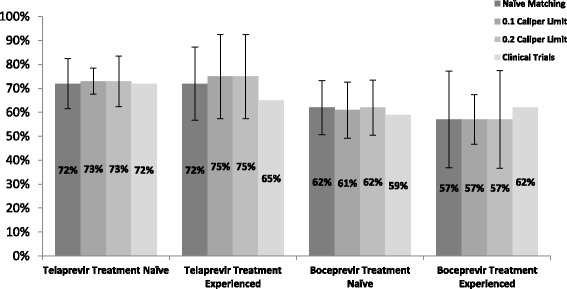
Comparison of the SVR24 rates between pivotal clinical trials and this study after adjusting for confounding

Pivotal TPV/PR and BOC/PR clinical trials were undertaken exclusively in treatment naïve or treatment experienced patients [40–45]. Comparison of the adjusted outcomes from this study with clinical trial data demonstrates that the adjusted SVR rates in both the TPV/PR and BOC/PR groups are comparable to those obtained in the clinical trials. In the TPV/PR treatment experience group, the adjusted SVR rates in this study demonstrate an improvement on the SVR rates obtained in the trials while the adjusted SVR rates for the BOC/PR treatment experienced are lower in this study when compared with the clinical trial data (Fig. 4).

### Comparison with real world studies

**Fig. 5:**
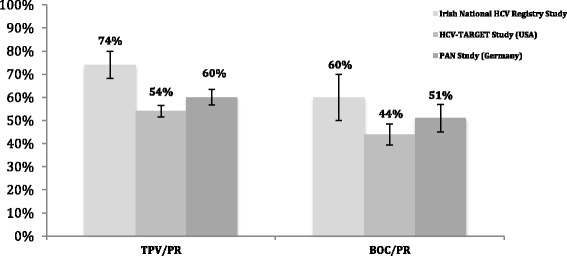
Comparison of the SVR24 rates between the Irish national registry and other international real world studies

A number of other real world studies assessing the effectiveness of TPV/PR and BOC/PR were initiated in the USA and Europe. We directly compared the SVR24 rates from our cohort with outcomes reported in the US-based HCV-TARGET study and the German-based PAN study (Fig. 5) [46, 47]. In the TPV/PR cohorts, a statistically significant difference was observed in the SVR24 rates between this study and both the HCV-TARGET and PAN studies (*p* < 0.05). In the BOC/PR cohorts, a statistically significant difference was observed between our study and the HCV-TARGET (*p* < 0.05) study but the difference between our study and the PAN study was not statistically significant (*p* = 0.141).

## Discussion

PS matching was applied to this dataset and is one of a number of tools that can be used to address the limitation due to confounding in non-randomised studies. It enables one to design and analyse an observational study so that it mimics some of the characteristics of a randomized controlled trial [48]. Random allocation allows conclusions to be made about the effect of treatment on outcomes by making direct comparisons between treated and untreated subjects, or between two treatment groups.

Prior to implementing PS matching there was an imbalance between confounding variables in the two treatment groups. While there is no international criterion that defines covariate imbalance, a standardised difference, between covariates of treated and untreated subjects, less than 0.1 is commonly accepted. This is taken to indicate a negligible difference in covariate balance between treatment groups [48]. Prior to matching, six of the nine covariates had a standardised difference greater then 0.1.

In order to improve the certainty surrounding our outcomes after PS matching, three approaches to PS matching were utilized. Naïve matching was the first approach to PS matching used. After matching, the standardised difference for each covariate was <0.1, indicating balance between the two groups. The logit of the propensity scores of the matched pairs was examined. The difference in logit of the final four pairs in each of the imputed datasets was considered poor (>0.2) and unacceptable. No significant impact on treatment effect was observed. The *p*-value for the odds ratio indicated that there was no statistically significant difference between TPV/PR and BOC/PR groups (*p*-value = 0.116), contradicting the crude odds ratio calculated before matching (*p*-value = 0.025). Given the unacceptability of a number of matched pairs, the matching was repeated, applying a caliper width limit of 0.1; the difference in logit of matches could not differ by more than 0.1. Ninety matched pairs were created. Standardised differences indicated balance between the TPV/PR and BOC/PR groups. Again, propensity score matching did not impact the SVR24 rate and the *p-*value indicated that there was no statistically significant difference between the two treatment groups (*p*-value = 0.097). A final, intermediate matching approach was implemented and a caliper width limit of 0.2 was applied. Ninety-one matched pairs were created and balance was observed between covariates in the two treatment groups. Analysis of the *p*-value indicated that there was no statistically significant difference in the odds of a SVR24 between the two groups and there was no impact on treatment effect.

After completing the three approaches to PS matching and adjusting for confounding in our data, balance in confounding variables between the two groups was observed. However, there was no difference in the SVR24 rates of the TPV/PR and BOC/PR groups prior to, and after PS matching (Fig. 3) While *p-*values after matching contrasted with the *p*-value before matching, the wide confidence intervals indicate significant uncertainty surrounding the outcomes.

Data reported in the Irish national HCV treatment registry confirms that the effectiveness in HCV treatment regimens in the Irish real world setting is generally comparable to the efficacy rates reported in clinical trials for similar patient cohorts, taking into account that historically, translation of outcomes from clinical trials is lower than in the clinical setting. Comparison of the SVR24 rates in the TPV/PR treatment naïve and treatment experienced subgroups in this study with the ADVANCE and ILLUMINATE treatment naïve, and REALIZE treatment experienced clinical trials indicate that the results in the real-world clinical setting are comparable, or better, than those reported in these trials [40–42]. The adjusted SVR24 rates in our TPV/PR treatment experienced group were between 7 and 10% higher than those observed in the REALIZE trial. The adjusted SVR24 rates in our BOC/PR treatment naïve group are comparable with outcomes from the SPRINT-1 and SPRINT-2 clinical trials but the results in the RESPOND trial are approximately 5% greater than the adjusted SVR24 rates in our BOC/PR treatment experienced group [43–45].

Additionally, the SVR outcomes were also compared with other international real world studies. The SVR24 rate from the Irish national HCV registry compared favourably with the outcomes from other international real world studies [46, 47]. Comparing key baseline demographics and HCV characteristics between the three studies demonstrated that our cohort was younger and had a higher proportion of males. However, our Irish cohort did include a lower proportion of patients with previous treatment experience while the proportion of patients with cirrhosis was lower than in the HCV-TARGET study but higher than the proportion in the PAN study [46, 47]. These demographic details suggest that the cohorts in the HCV-TARGET and PAN studies would be considered more difficult-to-treat than the Irish cohort and therefore, is reflected in the lower SVR24 rates reported in these studies.

## Conclusion

This study presents the effectiveness of triple therapy DAA regimens in a real-world clinical setting. PS matching is a useful tool for assessing real-world effectiveness while reducing the imbalance in confounding variables that exist in non-randomised studies. It generates adjusted outcome data that can be subsequently compared with clinical trial efficacy data. In this study, we conclude that there was no difference in the SVR24 rates of the TPV/PR and BOC/PR groups prior to, and after PS matching. The small sample size limits the conclusions that can be made about the effect of PS matching. While statistical significance was reported, the wide confidence intervals indicate significant uncertainty surrounding the outcomes. Propensity score adjustment remains a tool that can be applied to future analysis. However, we suggest, where possible, using a larger sample size in order to reduce the uncertainty around the outcomes.

## Additional file

Additional file 1: Table S1. Original confounding variables in dataset. **Table S2.** Confounding variables after inclusion of dummy variables. (DOCX 65 kb)

## Abbreviations

AE: Adverse event
BOC: Boceprevir
CI: Confidence interval
DAA: Direct-acting antiviral
GT: Genotype
HCV: Hepatitis C virus
IQR: Interquartile range
MCMC: Markov Chain Monte Carlo
OR: Odds ratio
PR: Pegylated-interferon and ribavirin
PS: Propensity score
PS: Propensity score
RCT: Randomised controlled trial
SVR: Sustained virological response
SVR2: Sustained virological response 24 weeks after treatment completion
TPV: Telaprevir
