# Supplementary material for: Effectiveness of triple therapy with direct-acting antivirals for hepatitis C genotype 1 infection: application of propensity score matching in a national HCV treatment registry
Source: BMC Health Serv Res. 2017 Apr 19;17:288. doi: 10.1186/s12913-017-2188-1 (PMC5395881; doi:10.1186/s12913-017-2188-1)
Supplement: Additional file 1: Table S1. — Original confounding variables in dataset. Table S2. Confounding variables after inclusion of dummy variables. (DOCX 65 kb) [file 12913_2017_2188_MOESM1_ESM.docx]

Table S1: Original confounding variables in dataset

| **Variable** | **Type** | **Value** |
| --- | --- | --- |
| Treatment experienced | Categorical | 1=Yes, 0=No |
| Presence of cirrhosis | Categorical | 1=Yes, 0=No |
| Age | Continuous |  |
| BMI | Continuous |  |
| Genotype | Categorical | 1=GT1, 2=GT1a, 3=GT1b |
| IL28B allele | Categorical | 1=CC, 2=CT, 3=TT |
| Baseline HCV>800,000 | Categorical | 1=Yes, 0=No |

Table S2: Confounding variables after inclusion of dummy variables

| **Variable** | **Type** | **Value** |
| --- | --- | --- |
| Treatment experienced | Categorical | 1=Yes, 0=No |
| Presence of cirrhosis | Categorical | 1=Yes, 0=No |
| Age | Continuous |  |
| BMI | Continuous |  |
| GT1 | Categorical | 1=Yes, 0=No |
| GT1a | Categorical | 1=Yes, 0=No |
| IL28B CT | Categorical | 1=Yes, 0=No |
| IL28B TT | Categorical | 1=Yes, 0=No |
| Baseline HCV>800,000 | Categorical | 1=Yes, 0=No |
